# Online Sensor Fault Detection Based on an Improved Strong Tracking Filter

**DOI:** 10.3390/s150204578

**Published:** 2015-02-16

**Authors:** Lijuan Wang, Lifeng Wu, Yong Guan, Guohui Wang

**Affiliations:** 1 College of Information Engineering, Capital Normal University, Beijing 100048, China; E-Mails: wlj0206@163.com (L.W.); guanYong@mail.cnu.edu.cn (Y.G.); wgh_boy@126.com (G.-H.W.); 2 Beijing Engineering Research Center of High Reliable Embedded System, Capital Normal University, Beijing 100048, China; 3 Beijing Key Laboratory of Electronic System Reliable Technology, Capital Normal University, Beijing 100048, China

**Keywords:** cubature Kalman filter, fault detection, strong tracking, sensor

## Abstract

We propose a method for online sensor fault detection that is based on the evolving Strong Tracking Filter (STCKF). The cubature rule is used to estimate states to improve the accuracy of making estimates in a nonlinear case. A residual is the difference in value between an estimated value and the true value. A residual will be regarded as a signal that includes fault information. The threshold is set at a reasonable level, and will be compared with residuals to determine whether or not the sensor is faulty. The proposed method requires only a nominal plant model and uses STCKF to estimate the original state vector. The effectiveness of the algorithm is verified by simulation on a drum-boiler model.

## 1. Introduction

With the wide application of sensors in production processes, sensors are used as the main devices of control systems to access information. Sensors are used in some systems to ensure the security, accuracy, and reliability of the system. Once these sensors are out of operation, they will affect the normal operation of the whole system, which may lead to serious consequences or disaster. Therefore, the detection of sensor faults is necessary.

Currently, most methods for detecting sensor faults are based on data-driven and model-based approaches. The advantage of the data-driven approach is that an accurate model is not needed, and the approach has strong adaptability. For example, expert systems and statistical analyses based on data-driven methods have been applied in fault detection with good results [1]. A method based on data fusion techniques using vectorized auto-regressive moving average models and multivariate orthogonal space transformations is proposed in this paper. Incremental neural networks and evolving fuzzy systems also have the ability to update sample-wise their parameters [2]. Although this method diagnoses faults only based on online recorded data streams, it requires a large database for operations that takes a long time and is computationally complex. In contrast, model-based methods have good real-time performance and do not require too much data [3]. A model-based approach is more powerful and shows a better performance when the process is well modeled. Model-based approaches detect faults by comparing the threshold with the residual generated by the true value and the estimated value.

Currently, Particle Filter (PF) [4] and Kalman Filter (KF) are the main model-based approaches for detecting faults. In order to ensure the accuracy of PF algorithm estimates, there must be a sufficient number of particles. A large number of samplings and resamplings are needed, and the calculations increase drastically with the growth of the space dimension. Because of the amount of computing time required, this method is not good at online fault diagnosis compared with KF. KF is a classic model-based method that has been widely used in fault detection and other fields [5]. The traditional KF algorithm predicts the next state for linear systems. Due to the KF method cannot be applied to increasingly complex systems, the Extended Kalman Filter (EKF) linearizes nonlinear systems by a first-order Taylor series expansion [6]. Moreover, the uncertainties of the model may lead to bias in the estimation process. Therefore, a fading factor is introduced in the Extended Kalman Filter to improve the accuracy of estimations when the model is not accurate, and this algorithm is called the Strong Tracking Filter (STF) [7]. To overcome the issue of EKF linearization errors a method called the Unscented Kalman Filter (UKF) is presented in [8]. A high-dimensional system, the sampling point of the UKF center right is negative, which leads to a non-positive definite covariance in the filtering process. This will affect the performance of the filter. However, when the system is a high-dimensional one, it is difficult for the UKF to achieve the desired effect. To improve the accuracy of estimates in high-dimensional systems, Arasaratnam [9] proposed a Kalman filter based on the cubature rule (CKF). Compared between the EKF and UKF, CKF has better nonlinear approximation performance, numerical accuracy, and stability, and is relatively simple to achieve. However, when there are many differences between the model parameters and process parameters, the precision of the estimations of all of the Kalman filters will greatly decrease, and even diverge.

Prediction accuracy will directly affect the quality of the residuals and the accuracy of fault detection. In view of this, we propose a new method that uses cubature rules instead of the Jacobian matrix in a Strong Tracking Filter to generate steady residuals in fault-free cases. The proposed method combines the advantages of CKF and STF to produce more accurate estimated values and more stable residuals in fault-free cases. Setting up a suitable threshold to compare with the residual, a sensor fault is considered to have occurred when the residual exceeds the threshold. Thus, sensor fault detection is realized. The above approach to detecting sensor faults is based on the assumption that, in the process, the actuator is fault free and the system is fault free.

This paper is organized as follows: in Section 2 a mathematical formulation of the system is described and the assumptions and needs of the system are given. In Section 3 an algorithm for fault detection is proposed. A simulation on a drum-boiler model is provided to verify the effectiveness of the algorithm. The results of simulation are discussed in Section 4. Conclusions are presented in Section 5.

## 2. Problem Formulation

### 2.1. Nonlinear System Model

The system model is described using the following state space model:

(1) $$x_{k}=\;f\left(x_{k-1},u_{k-1}\right)\;+w_{k-1}$$

(2) $$y_{k}=h\left(x_{k},u_{k}\right)\;+v_{k}$$

where *x_k_* ∈ ℝ*_n_*_×1_ is the state vector, *y_k_* ∈ ℝ*_m_*_×1_ is the outputs vector, *u_k_* ∈ ℝ*_l_*_×1_ is the control vector, and *w_k_* and *v_k_* are noise sequence of the process and measurement, respectively. The mean and covariance matrices of *Q_k_* and *R_k_*, respectively. *f*(·) and *h*(·) are known functions related to the system.

### 2.2. Residual Generation

We assume that the state estimated value and the outputs estimated value based on the mathematical model of the system are *x̂_k_* and *ŷ_k_*, and that *x̂_k_* and *ŷ_k_* can be estimated according to the KF. We consider the outputs of system *y_k_* to be the true value *ŷ_k_*. We can define r(·) as the residual. It is a signal symptom to judge whether or not the system is a failure. Its form is described as follows:

(3) $$\text{r}\left(\text{k}\right)=y_{k}-{\hat{y}}_{k}$$

Under fault-free conditions, the residual is close to zero. This signal should deviate from zero and exceed a predetermined value when a fault occurs. When a fault occurs, the estimated value *ŷ_k_* will take the place of the failure value to ensure the normal operation of the system.

### 2.3. Fault Detection

**Figure 1.**
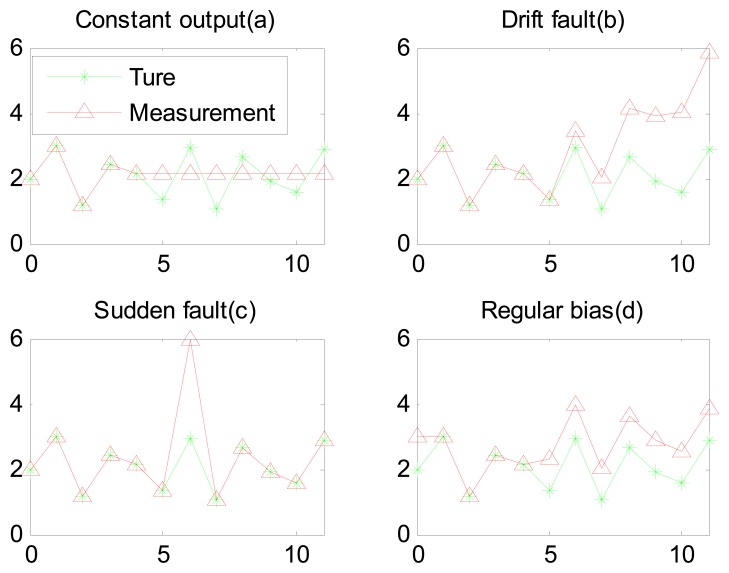
Common sensor fault types: (**a**) Constant output fault; (**b**) Drift fault; (**c**) Sudden fault; (**d**) Regular bias.

We consider a process that is actuator fault free and system fault free. The goal is to detect sensor failures. Sensor faults can be summarized as falling under four categories, as illustrated in Figure 1 [10]. We consider that only one type of fault at a time occurs on a sensor.

## 3. Algorithm for Fault Detection

In this section a cubature rule is used in the process of estimating the state of a strong STF, and in generating the residual to judge whether or not a fault has occurred. If a fault has occurred, an alarm signal will be generated. We designed a series of filters to generate residuals for detecting sensor faults.

### 3.1. Filter Design

A filter is designed to produce a residual value. A steady residual value is more advantageous for detecting faults. The proposed approach has simple calculation advantages compared with UKF and EKF. CKF avoids the need to solve the Jacobian matrix in EKF. It has fewer sampling points than UKF, so CKF has more advantages in a high-dimensional system. In [11] a method involving the introduction of a forgetting factor for the smooth treatment of drifts in data streams is adopted, and a good performance is achieved. The STF is a classic method often used to estimate the state of the system to complete the detection of faults [12]. The fading factor *λ_k_*_+1_ is introduced in the time update process to obtain an accurate estimated value when the parameters of the model do not match. The fading factor is obtained by forcing a residual orthogonal. The proposed method has the advantages of STF and CKF. The STF time update and measurement update steps are given as follows:

Time update:

The predicted *x̂_k_*_+1|_*_k_* and associated covariance *P_k_*_+1|_*_k_* are calculated as follows:

(4) $${\hat{x}}_{k+1|k}=\;A_{k}{\hat{x}}_{k|k}$$

(5) $$p_{k+1|k}=\lambda_{k+1}\;A_{k}P_{k|k}A_{k}^{T}+Q_{k}$$

where:

$$A_{k}=\;\frac{\partial f\left(x_{k},u_{k}\right)}{\partial x_{k}}$$

$$C_{k}=\;\frac{\partial h\left(x_{k},u_{k}\right)}{\partial x_{k}}$$

Gain:

(6) $$H_{k+1}=P_{k+1|k}C_{k+1}^{T}{\left(C_{k+1}P_{k+1|k}C_{k+1}^{T}+R_{k+1}\right)}^{-1}$$

Measurement update:

The predicted measurement *x̂_k_*_+1|_*_k_*_+1_ and associated covariance *P_k_*_+1|_*_k_*_+1_ are calculated as follows:

(7) $${\hat{x}}_{k+1|k+1}=\;A_{k+1}{\hat{x}}_{k+1|k}+H_{k+1}\left(y_{k+1}-C_{k+1}{\hat{x}}_{k+1|k}\right)$$

(8) $$P_{k+1|k+1}=\left(I-H_{k+1}C_{k+1}\right)P_{k+1|k}$$

The fading factor *λ_k_*_+1_ can be obtained as follows:

(9) $$\lambda_{k+1}=\;\{\begin{array}{c} \lambda_{0}\;\left(\lambda_{0}\geq 1\right)\\ 1\;\left(\lambda_{0}<1\right) \end{array}$$

(10) $$\lambda_{0}=\frac{tr\left[N_{k+1}\right]}{tr\left[M_{k+1}\right]}$$

(11) $$N_{k+1}=\;V_{k+1}-\;\beta R_{k+1}-C_{k+1}Q_{k}\;C_{k+1}^{T}$$

(12) $$M_{k+1}=\;C_{k+1}A_{k+1}P_{k+1|k+1}\;A_{k+1}^{T}C_{k+1}^{T}$$

(13) $$V_{k+1}=\;\{\begin{array}{c} r_{1}r_{1}^{T}\;\left(k=1\right)\\ \frac{\rho V_{k}+r_{k}r_{k}^{T}}{1+\rho }\;\left(k>\;1\right) \end{array}$$

*tr*(·) is the tracing operation, *ρ* is the forgetting factor of the residual sequence, *ρ* = 0.95, *β* is the weakening factor, generally *β* > 0, and the value of the *β* and *ρ* are selected based on experience.

For strong nonlinear systems, the STF often introduces large linear errors. It linearizes nonlinear systems by a first-order Taylor series expansion in the process of estimating. We introduced cubature rules into the process of STF estimates. Arasaratnam [9] proposed the use of the CKF algorithm according the cubature rules selecting the 2n point set (*ξ_i_*, *ω_i_*) with the same approximate weight as the integral value:

(14) $$\text{J}\left(f\right)=\sum_{i=1}^{2n}\omega_{i}\;f\left(\xi_{i}\right)$$

where $\xi_{i}=\sqrt{m/2}$, *ω_i_*=1/*m*, *n* stands for the system state vector dimension; *m*=2*n*.

Initialize

$${\hat{x}}_{0|0}=E\left[x_{0}\right]$$

$$p_{0|0}=E\left[\left(x_{0}-{\hat{x}}_{0|0}\right){\left(x_{0}-{\hat{x}}_{0|0}\right)}^{T}\right]$$

Time update:

According to the state model transforming we can obtain cubature points *x̃*_*i*,*k*__+1|_*_k_*. Using cubature points, we predict the state and the error covariance:

(15) $${\tilde{x}}_{i,k+1|k}=f\left(x_{i,k+1|k},u_{k}\right)+w_{k}$$

(16) $${\hat{x}}_{k+1|k}=\;\frac{1}{m}\sum_{i=1}^{m}{\tilde{x}}_{i,k+1|k}+w_{k}$$

(17) $$p_{k+1|k}=\;\frac{1}{m}\lambda_{k+1}\sum_{i=1}^{m}{\tilde{x}}_{i,k+1|k}{\tilde{x}}_{i,k+1|k}^{T}-{\hat{x}}_{k+1|k}{\hat{x}}_{k+1|k}^{T}+Q_{k}$$

Measurement update:

According to the state model transforming we can obtain cubature points *ỹ*_*i*,*k*__+1|_*_k_*. Using cubature points we obtain prediction outputs and the prediction covariance:

(18) $${\tilde{y}}_{i,k+1|k}=h\left(x_{i,k+1|k}\right)+v_{k}$$

(19) $${\hat{y}}_{i,k+1|k}=\;\frac{1}{m}\sum_{i=1}^{m}{\tilde{y}}_{i,k+1|k}+v_{k}$$

(20) $$p_{yy,k+1|k}=\;\frac{1}{m}\sum_{i=1}^{m}{\tilde{y}}_{i,k+1|k}{\tilde{y}}_{i,k+1|k}^{T}-{\hat{y}}_{k+1|k}{\hat{y}}_{k+1|k}^{T}+R_{k}$$

(21) $$p_{xy,k+1|k}=\;\frac{1}{m}\sum_{i=1}^{m}{\tilde{x}}_{i,k+1|k}{\tilde{y}}_{i,k+1|k}^{T}-{\hat{x}}_{k+1|k}{\hat{y}}_{k+1|k}^{T}$$

Computational gain:

(22) $$H_{k+1}=\;p_{xy,k+1|k}\;p_{yy,k+1|k}^{-1}$$

Optimal estimate:

Update the estimation of the state and the associated covariance:

(23) $${\hat{x}}_{k+1|k+1}={\hat{x}}_{k+1|k}+H_{k+1}\left(y_{k+1}-{\hat{y}}_{k+1|k}\right)$$

(24) $$p_{k+1|k+1}=\;p_{k+1|k}-\;H_{k+1}p_{yy,k+1|k}H_{k+1}^{T}$$

However, according to the literature [13], the fading factor can be obtained in the case of estimations using cubature rules. Through the Equations (9), (10), (11), (12), and (13) we obtain the fading factor for STCKF, and *N_k_*_+1_ and *M_k_*_+1_ will be replaced by the following:

(25) $$N_{k+1}=\;V_{k+1}-\;R_{k+1}-{\left[p_{xy,k+1|k}\right]}^{T}{\left[p_{k+1|k}\right]}^{-1}Q_{k}\;{\left[p_{k+1|k}\right]}^{-T}p_{xy,k+1|k}$$

(26) $$M_{k+1}=\;p_{yy,k+1|k}-V_{k+1}+N_{k+1}$$

### 3.2. Setting a Threshold

The residual determines fault status by applying a decision-making function. In [14] the authors set up an adaptable threshold that is incrementally/decrementally updated over a sliding window. It reduced the false alarm and missed alarm rates. In [15] an adaptable threshold value by using fuzzy logic and the weighted average method, which is more effective for graded fault detections, was set up. However, because the residual obtained by STCKF is sufficiently stable, we used a simple method of setting thresholds that can have a good effect on detecting faults. The residual is the difference between the true value and the estimate value. The fault-free residual includes the noise and estimate error. Due to the residual includes fault information in fault case, the residual in fault case will beyond the fault-free residual. We use the statistical properties of residual in fault-free case. The fixed threshold function can be described as follows [16]:

(27) $$r_{k}^{i}=y_{k}^{i}-{\hat{y}}_{k}^{i}$$

where $r_{k}^{i}$ represents the residual of the i-th sensor at time k in a fault-free case:

(28) $${\bar{r}}^{i}=\frac{1}{m}\sum_{k=1}^{m}|r_{k}^{i}|$$

(29) $$\sigma_{i}^{2}=\frac{1}{m}\sum_{j=1}^{m}{\left(\left|r_{j}^{i}\right|-\bar{r^{\iota }}\right)}^{2}$$

where *r̄^i^* is the i-th sensor's mean of the fault-free residual, and $\sigma^{i}\sigma_{i}^{2}$ is the i-th sensor's variance of the fault-free residual.

If $\left|r_{k}^{i}\right|\leq {\bar{r}}^{i}+\sigma^{i}\left|r_{k}^{i}\right|<\bar{r^{\iota }}+b\sigma_{i}^{2}$, this proves that this is a fault-free case. At the same time, alarm signal *F_k_* will be set to 0. If $\left|r_{k}^{i}\right|$ exceeds the threshold, this proves that this is a case of fault. The alarm signal will be set to 1. This method is simple and effective for the fault detection.

## 4. Experimental Analysis

In this section the effectiveness of the proposed algorithm will be validated on a drum-boiler model. We will verify the effect of fault detection in three cases of failure, respectively. Finally, the test results will be analyzed.

### 4.1. Model Description

A drum-boiler model will be cited to validate that the proposed algorithm is effective. It is a subsystem of a thermal power plant unit where the separation of water and steam takes place. More details about the construction and operation of a drum-boiler are given in [17]. The nonlinear state-space model used in the experiment was taken from [4]. Its parameters were identified at a 160 MW oil-fired Synvendska Kraft AB Plant, and the model is expressed as follows:

(30) $${\dot{x}}_{1}=-0.0018u_{2}x_{1}^{\frac{9}{8}}+0.9u_{1}-0.15u_{3}$$

(31) $${\dot{x}}_{2}=(0.073u_{3}-0.016)x_{1}^{\frac{9}{8}}-0.1x_{2}$$

(32) $${\dot{x}}_{3}=\left(141u_{3}-\left(1.1u_{2}-0.19\right)x_{1}\right)/85$$

(33) $$y_{1}=x_{1}$$

(34) $$y_{2}=x_{2}$$

(35) $$y_{3}=0.05\left(0.13073x_{3}+100a_{cs}+\frac{q_{e}}{9}-67.975\right)$$

(36) $$a_{cs}=\frac{\left(1-0.001538x_{3}\right)\left(0.8x_{1}-25.6\right)}{\left(1.0394-0.0012304x_{1}\right)x_{3}}$$

(37) $$q_{e}=\left(0.854u_{2}-0.147\right)x_{1}+45.59u_{1}-2.514u_{3}-2.096$$

where *x*_1_ and *x*_2_ are output variables of the drum pressure (kg/cm^2^) and electrical output (MW) respectively, *x*_3_ is the fluid density (kg/m^3^), and *y*_3_ is the drum water level (m); and where *u*_1_ and *u*_3_ are the fuel and feedwater flows in T/hr respectively, *u*_2_ is the control value position; *a_cs_* is the steam quality; and *q_e_* is the evaporation rate.

The paper [4] used PF to estimate the states of this nonlinear model. Because PF requires a large number of samplings and resamplings, the calculation drastically increases with the growth of the space dimension. In terms of computing time, this method is not good at diagnosing online fault compared with the STCKF.

For present fault detection, a discrete-time model is obtained from Equations (30), (31), and (32). The sampling period is *T_s_* = 5 s. The values obtained from the model are actual output values, and the values obtained by the filter are estimated values.

### 4.2. Simulation Results for Faulty Cases

**Figure 2.**
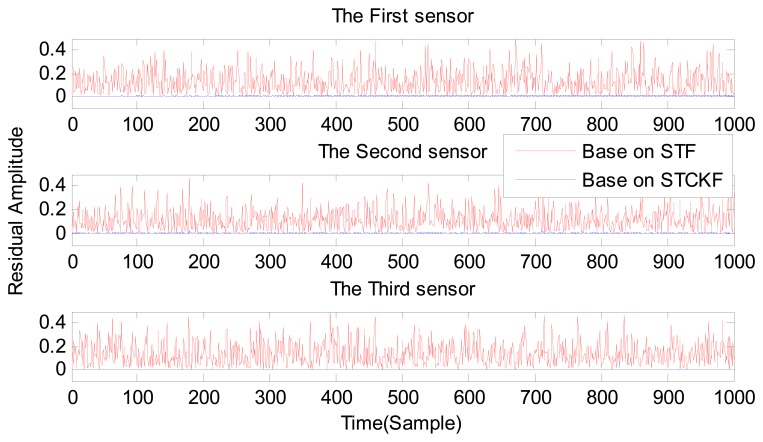
The sensor outputs residual in a fault-free case obtained by STCKF, UKF, and STF.

Under fault-free conditions, the absolute value of three residuals, obtained through the STCKF, UKF, and STF, respectively, are compared in Figure 2. Comparing the residuals obtained through the three different algorithms, it is evident that STCKF has the highest accuracy. Residuals obtained by STCKF under a fault-free condition are very smooth. The STCKF can reduce rates of false alarms and missed alarms in the fault detection process. Because the constant output failure is very easy to diagnose, we only test the other three types of fault. We verify the effectiveness of the algorithm under three cases of fault. The deviation between the actual output values and the predicted values is greater as the fault level improves. Therefore, greater residual amplitudes are generated and faults will be detected more easily. Our simulation faults deviated 5%, 10%, and 20% from the normal measurements.

In case (1) a sudden fault occurs in the first sensor at the *t* = 120 output. The sudden fault deviated 5%, 10%, and 20% from the normal measurement in the first sensor output, respectively.

In case (2) a drift fault in the first sensor deviated 5%, 10%, and 20% from the normal measurement at the *t* ≥ 120 output. In case (3) a regular bias fault occurs in the first sensor, which is 5%, 10%, and 20% from the normal measurement at the *t* ≥ 120 output. The same conditions as those in cases (1)–(3) are simulated in the second and third sensors.

**Figure 3.**
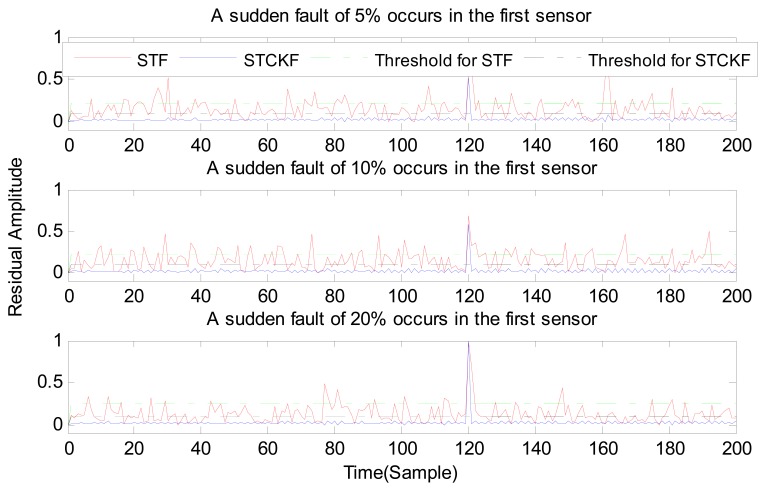
A sudden fault occurs only in the first sensor based, on STCKF, UKF, and STF, at *t* = 90.

**Figure 4.**
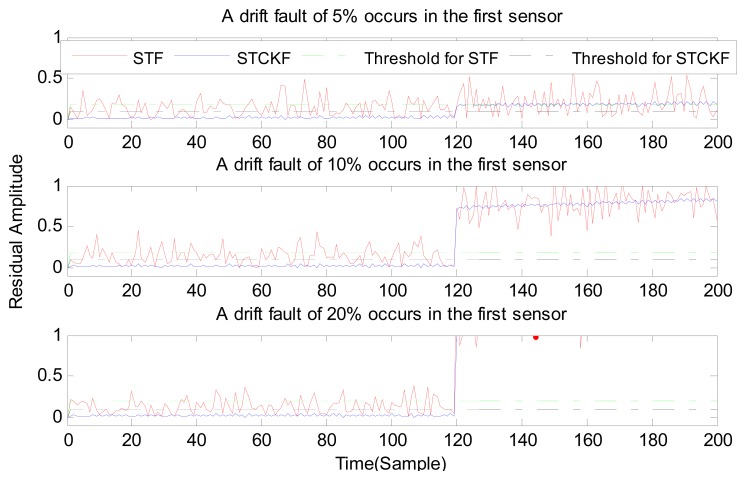
A drift fault occurs only in the first sensor, based on STCKF, UKF, and STF, at *t* ≥ 120.

**Figure 5.**
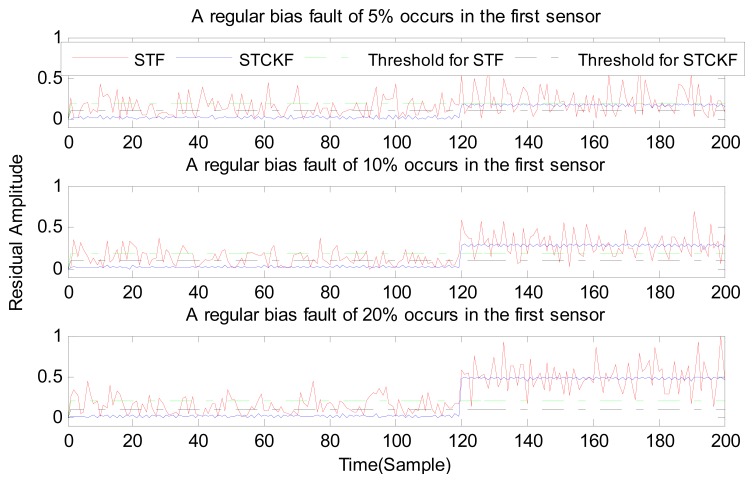
A regular bias fault occurs only in the first sensor, based on STCKF, UKF, and STF.

**Figure 6.**
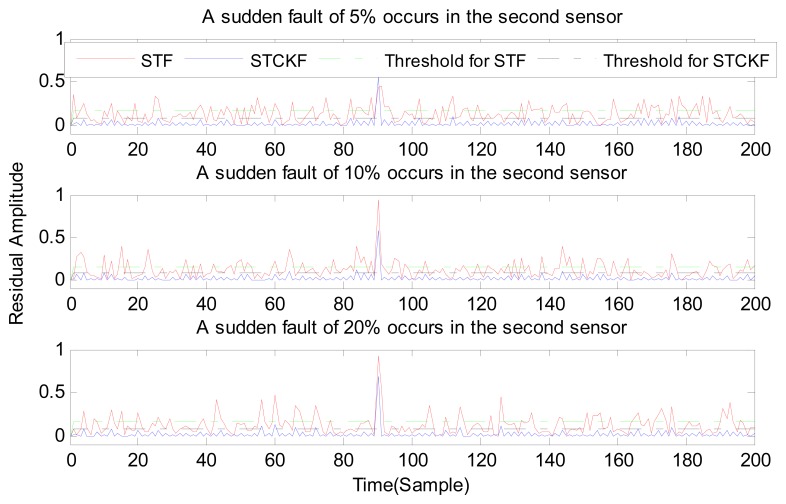
A sudden fault occurs only in the second sensor, based on STCKF, UKF, and STF, at *t* = 90.

**Figure 7.**
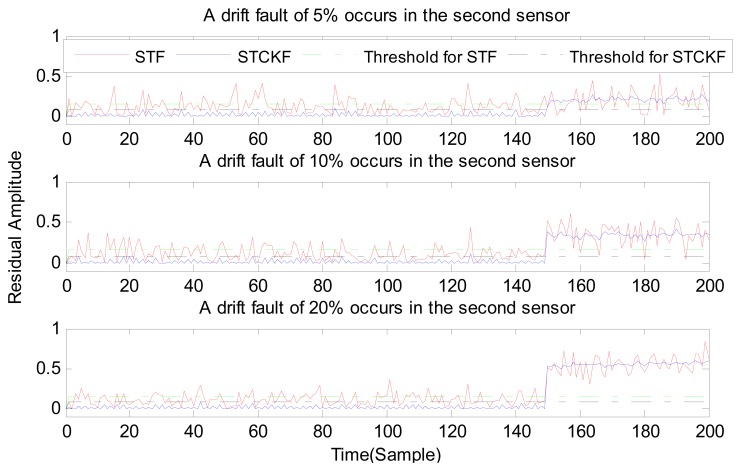
A drift fault occurs only in the second sensor, based on STCKF, UKF, and STF, at *t* ≥ 150.

**Figure 8.**
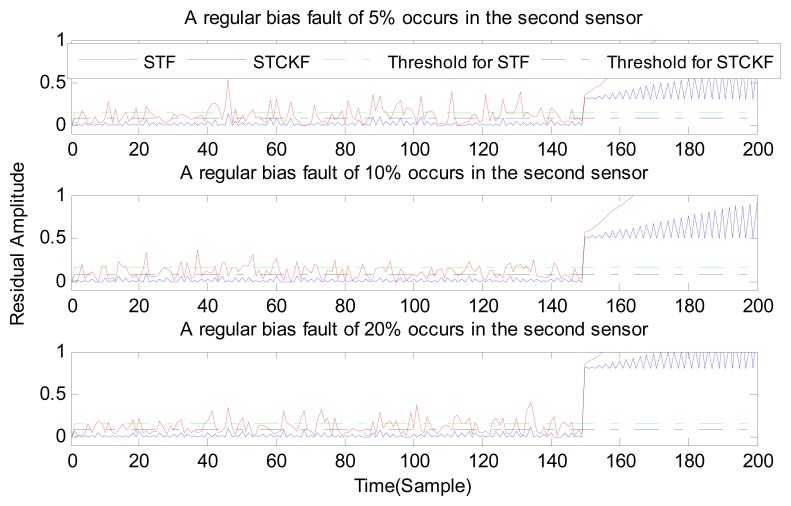
A regular bias fault occurs only in the second sensor, based on STCKF, UKF, and STF, at *t* ≥ 150.

**Figure 9.**
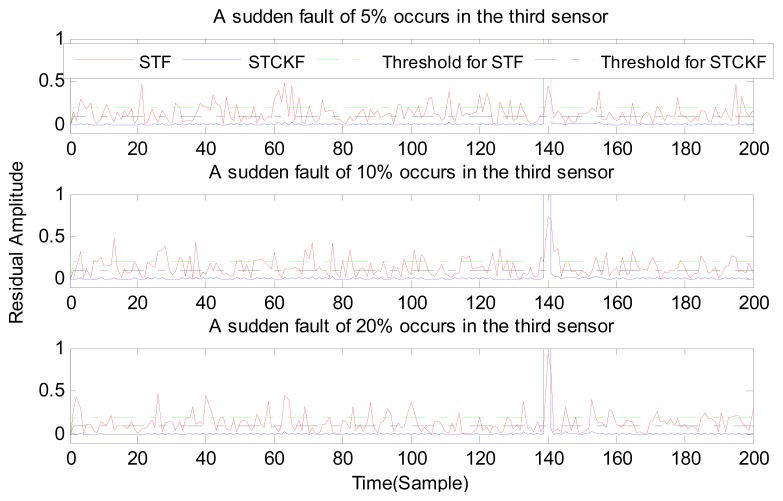
A sudden fault occurs only in the third sensor, based on STCKF, UKF and STF, at *t* =140.

**Figure 10.**
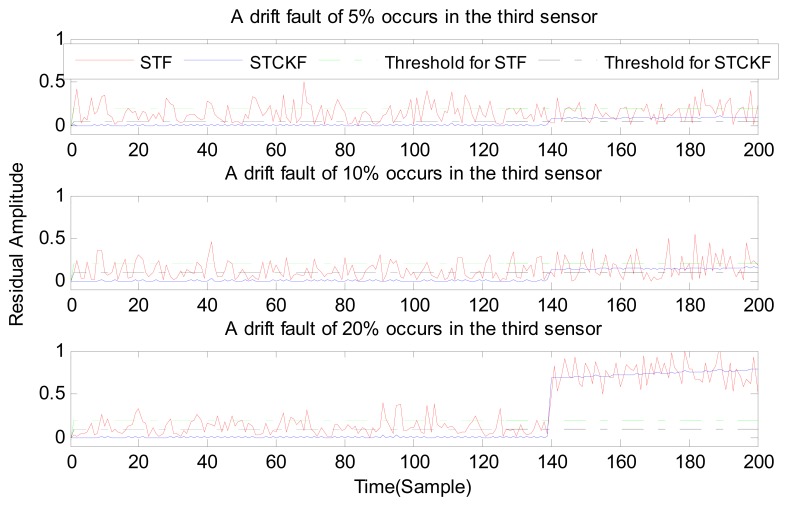
A drift fault occurs only in the third sensor, based on STCKF, UKF, and STF, at *t* ≥ 140.

**Figure 11.**
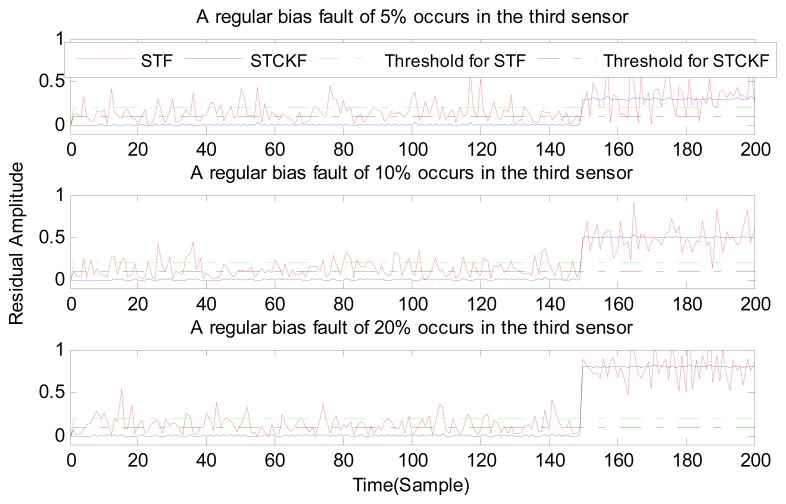
A regular bias fault occurs only in the third sensor, based on STCKF, UKF, and STF, at *t* ≥ 150.

As shown in Figures 3, 4, 5, 6, 7, 8, 9, 10 and 11, the method proposed has good forecasting performance. A smooth residual generated by STCKF greatly reduced the false alarm rates and missed alarm rates. STCKF has better performance than STF in fault detection. The STF method produces larger errors than the other methods in the prediction process for strong nonlinear systems, and this has a negative effect on fault detection. In the case of small regular bias faults and drift faults, as shown in the first picture in Figure 10, the STF method cannot even produce effective residuals. Because the residual value obtained by STCKF is smooth under fault-free conditions and exhibits obvious changes when the sensors come across failure, we can use a simple method to set a fixed threshold for fault detection and achieve good implementation.

To show the performance of the fault detection results between the different methods, we performed a statistical analysis. The rates of missed alarms or false alarms in the detection of faults are shown in Table 1. The results listed in Table 1 are the statistical results of 200 consecutive occurrences of fault.

From Table 1 we see that the trend is that the rate of false alarms will not increase as the size of the fault increases. The proposed method performs better than STF. With the proposed method, when the size of the fault is greater than 5% of the output value, the rate of missed alarms is approximately zero.

## 5. Conclusions

In this paper, a new approach to online sensor fault detection was proposed. Moreover, this approach offers the advantage of accurate and simple calculations. With the aim of obtaining more accurate estimates, which can make the residuals smooth, thereby reducing the rates of missed alarms and false alarms, a cubature rule was introduced in the STF. For strong nonlinear systems, the proposed algorithm offers a great improvement in accuracy. It generated smooth residuals in fault-free cases, which made the detection of faults accurate. To evaluate the proposed approach, it was applied to a nonlinear model of a drum-boiler. The results of the simulation confirmed that this method is more efficient at online sensor fault detection. The case of the faults of different, multiple interrelated sensors will be the focus of the next stage of research.

## Figures and Tables

**Table 1.** Detection rates with different types of fault.

**Faulty Sensor**	**Fault Type**	**Missed Alarm (%)**			**False Alarm (%)**		

		5%	10%	20%	5%	10%	20%
The first sensor	Regular Bias	-	-	-	-	-	-
	STCKF	0.20	0	0	1.27	1.31	1.20
	STF	46.7	24.9	3.84	28.8	27.4	22.2
	Drift fault	-	-	-	-	-	-
	STCKF	0	0	0	1.30	1.27	0.33
	STF	14.5	0	0	28.6	27.1	26.1
	Sudden fault	-	-	-	-	-	-
	STCKF	0	0	0	1.27	1.60	1.79
	STF	0	0	0	22.7	22.2	22.8

The second sensor	Regular Bias	-	-	-	-	-	-
	STCKF	0	0	0	0.67	0.68	0.67
	STF	0.14	0	0	32.0	30.8	30.1
	Drift fault	-	-	-	-	-	-
	STCKF	0	0	0	0.67	0.67	0
	STF	35.8	10.6	0.50	30.4	30.4	30.2
	Sudden fault	-	-	-	-	-	-
	STCKF	0	0	0	0.39	0.50	0.49
	STF	0	0	0	31.1	31.1	31.1

The third sensor	Regular Bias	-	-	-	-	-	-
	STCKF	0	0	0	0.67	0.67	0.67
	STF	28.3	4.60	0.04	25.4	24.9	24.8
	Drift fault	-	-	-	-	-	-
	STCKF	0	0	0	0.72	0.72	0.72
	STF	3.46	1.72	0	25.1	24.6	25.3
	Sudden fault	-	-	-	-	-	-
	STCKF	0	0	0	0.39	0	0
	STF	0	0	0	15.8	15.7	15.5

## References

[1] Serdio F., Lughofer E., Pichler K., Buchegger T., Efendic H. (2014). Residual-Based Fault Detection Using Soft Computing Techniques for Condition Monitoring at Rolling Mills. Inf. Sciences.

[2] Lughofer E. (2011). Evolving Fuzzy Systems. Methodologies, Advanced Concepts and Applications.

[3] Rajaraman S., Hahn J., Mannan M.S. (2006). Sensor fault diagnosis for nonlinear processes with parametric uncertainties. J. Hazard. Mater..

[4] Tadic P., Ðurovic Z. (2014). Particle filtering for sensor fault diagnosis and identification in nonlinear plants. J. Process Control.

[5] Sun B., Luh P.B., Jia Q.-S., O'Neill Z. (2014). Building energy doctors: An SPC and Kalman filter-based method for system-level fault detection in HVAC systems. Trans. Autom. Sci. Eng..

[6] Rjaraman S. (2006). Robust Model-Based Fault Diagnosis for Chemical Process Systems. PhD Thesis.

[7] Sun L., Dong J., Li D., Zhang Y. (2014). Model-Based Water Wall Fault Detection and Diagnosis of FBC Boiler Using Strong Tracking Filter. Math. Probl. Eng..

[8] Wan E.A., van der Merwe R. (2000). The unscented Kalman filter for nonlinear estimation.

[9] Arasaratnam I., Haykin S. (2009). Cubature Kalman filters. IEEE Trans. Autom. Control.

[10] Zhu D.-Q., Chen L., Liu Q. (2009). Sensor fault diagnosis and fault-tolerant control method of underwater vehicles. Control Decis..

[11] Shaker A., Lughofer E. (2014). Self-Adaptive and Local Strategies for a Smooth Treatment of Drifts in Data Streams. Evol. Syst..

[12] Zhou D.H., Xi Y.G., Zhang Z.J. (1990). Suboptimal fading extended Kalman filtering for nonlinear systems. Control Decis..

[13] Wang X.X., Zhao L., Xia Q.X. (2010). Strong tracking filter based on unscented transformation. Control Decis..

[14] Serdio F., Lughofer E., Pichler K., Buchegger T., Pichler M., Efendic H. (2014). Fault Detection in Multi-Sensor Networks based on Multivariate Time-Series Models and Orthogonal Transformations. Inf. Fusion.

[15] Ruan W., Qing H., Cong L., Xu P. (2012). Improved residual χ^2^ inspection in integrated navigation fault detection of application. Electron. Meas. Technol..

[16] Zarei J., Shokri E. (2014). Robust sensor fault detection based on nonlinear unknown input observer. Measurement.

[17] Aström K., Bell R. (2000). Drum-boiler dynamics. Automatica.

